# Epigenetic Modifications at the Center of the Barker Hypothesis and Their Transgenerational Implications

**DOI:** 10.3390/ijerph182312728

**Published:** 2021-12-02

**Authors:** Rebecca Jean Ryznar, Lacie Phibbs, Lon J. Van Winkle

**Affiliations:** 1 Molecular Biology, Department of Biomedical Sciences, Rocky Vista University, Parker, CO 80134, USA; 2 College of Osteopathic Medicine, Rocky Vista University, Parker, CO 80134, USA; lacie.phibbs@rvu.edu; 3 Department of Medical Humanities, Rocky Vista University, Parker, CO 80134, USA; lvanwinkle@rvu.edu

**Keywords:** Barker hypothesis, transgenerational epigenetics, amino acid transporters, exosomes, short tRNA fragments

## Abstract

Embryo/fetal nutrition and the environment in the reproductive tract influence the subsequent risk of developing adult diseases and disorders, as formulated in the Barker hypothesis. Metabolic syndrome, obesity, heart disease, and hypertension in adulthood have all been linked to unwanted epigenetic programing in embryos and fetuses. Multiple studies support the conclusion that environmental challenges, such as a maternal low-protein diet, can change one-carbon amino acid metabolism and, thus, alter histone and DNA epigenetic modifications. Since histones influence gene expression and the program of embryo development, these epigenetic changes likely contribute to the risk of adult disease onset not just in the directly affected offspring, but for multiple generations to come. In this paper, we hypothesize that the effects of parental nutritional status on fetal epigenetic programming are transgenerational and warrant further investigation. Numerous studies supporting this hypothesis are reviewed, and potential research techniques to study these transgenerational epigenetic effects are offered.

## 1. Introduction

Barker and associates postulated that variations in fetal programming can account for undesirable health outcomes in adults, which is now known as the Developmental Origins of Health and Disease (DOHaD) or the Barker hypothesis [1,2,3]. The overarching notion of this theory is that unfavorable intrauterine conditions can lead to metabolic disease in adulthood. Conditions that alter metabolic programming in early embryos govern more permanent changes in metabolic signaling pathways and, subsequently, abnormal fetal and placental growth and development [1]. Nutrient deficiencies can stunt early fetal growth, and intrauterine over-nutrition, such as in obesity or gestational diabetes, can trigger changes in fetal metabolic programming associated with poor health outcomes in offspring. 

## 2. Fetal Growth and Importance of Nutrition: Barker Hypothesis and DOHaD Model

The DOHaD theory can account for the strong association of ischemic heart disease mortality rates in 1968–1978 with infant mortality rates in the same locations in 1921–1925 [2]. Since low birthweight was the most common cause of infant death at the time, it was hypothesized that surviving low birth weight (LBW) infants had higher risks of ischemic heart disease later in life. In the years to follow, Barker and colleagues continued to share epidemiologic data that showed a strong association between LBW and ischemic heart disease [3]. In 1992, the ‘thrifty phenotype’ hypothesis proposed that type 2 diabetes mellitus may develop due, in part, to fetal adaptation to intrauterine hypoglycemia [4]. Barker and colleagues further hypothesized that fetal adaptations to poor nutrition resulted in lasting changes of the sensitivity and response to hormones leading to increased adult disease risk [5]. These observations have been validated in multiple populations, including data from the Dutch Famine in 1944, which showed that those who experienced nutritional scarcity showed a higher incidence of cardiovascular disease and type 2 diabetes [6]. Several other associations between low birthweight and adult disease were discovered through epidemiological studies, including insulin resistance, cardiovascular disease, hypertension, and stroke [7]. This review explores various mechanisms by which nutrition can alter the early epigenome and how these alterations can be passed down to subsequent generations. 

Recently, a study of 237,631 individuals utilizing Mendelian randomization demonstrated a causal link between low birth weight and increased risk of coronary artery disease and type 2 diabetes [8]. Additionally, using Mendelian randomization, causal connections have been established between high birth weight, a higher BMI in adulthood [8], and increased incidence of atrial fibrillation [9]. These data provide considerable evidence for a relationship between birth weight and various diseases in adulthood. 

In a recent systematic review, de Mendonça and associates examined 64 studies and found an association between LBW, SGA, and preterm birth with cardiometabolic disorders, glycidic metabolism disorders, and increased risk of metabolic syndrome [10]. In their meta-analysis of 18 articles, there was a positive association between LBW and incidence of cardiometabolic disease, metabolic syndrome, and type 2 diabetes in adulthood. 

While the mechanism underlying these links is yet to be fully elucidated, recent research is focused on genetic and epigenetic causes [11]. Epigenetics is the inheritance of variation in gene expression without changes in the DNA nucleotide sequence. Chemical modifications of DNA and histones constitute the epigenome and are regulated by metabolic pathways, transporters, and diet.

The Agouti mouse model showed a link between maternal diet, epigenetic changes, and fetal outcomes. Specifically, methyl supplementation of the mother’s diet during pregnancy resulted in a shift of offspring phenotype away from the yellow phenotype, linked with adult-onset diabetes and obesity, toward the brown phenotype that is free of these comorbidities, owing to epigenetic modifications [12]. In nonhuman primates, an effect of maternal diet on epigenome changes has also been demonstrated. Aagaard-Tillery et al. importantly identified that in utero exposure to a high-fat maternal diet induced fetal hepatic histone H3 acetylation [13]. Such epigenetic changes result in alteration to normal fetal growth and may explain the link between LBW and adult disease.

## 3. Physiologic Mechanisms Linking Low Birth Weight and Adult Disease

Normal fetal growth depends on oxygen and nutrient delivery via the placenta. When this delivery is compromised, the fetus responds by preserving certain processes, such as brain growth, at the cost of the fetus’s overall size. To spare vital organs, the fetus increases vasoconstriction of peripheral blood vessels [14] and cortisol production [15], thereby shunting blood to the brain. Hypoxia results from known sources such as placental abnormalities but may also be the result of reduced nutrient delivery or overnutrition. For example, fetal adaptation to reduced nutrients has been demonstrated to increase hypoxia signaling in the liver via increased fetal hepatic insulin-like growth factor binding protein-1 [16,17]. Placentas from mice fed a high-fat diet also have shown impaired vascularization and hypoxia [18]. Additionally, there is a positive association between maternal obesity and erythropoietin concentration in cord blood, suggesting that chronic fetal hypoxia is occurring in the pregnancies of obese women [19]. Such hypoxia, of course, leads to slower fetal growth restriction and lower birth weight [20]. 

To understand the pathophysiology behind LBW and adult disease, it is important to first discuss this infant classification. LBW is defined as birth weight < 2.5 kg and encompasses constitutionally small, premature, small for gestational age (SGA), and fetal/intrauterine growth-restricted (FGR/IUGR) infants. Recent consensus defines SGA as a fetus born with a birth weight <10th percentile for gestational age or more than two standard deviations below the population mean. This definition, however, does not distinguish between SGA infants who are small due to growth-restriction and those who are constitutionally small. FGR requires additional criteria of estimated fetal weight or abdominal circumference less than the third percentile for early FGR (<32 weeks) or less than the 10th percentile for late FGR (≥32 weeks) [21]. While FGR is inherently pathological, SGA may not be depending on underlying etiology. Regardless of its underlying cause, LBW results from fetal malnutrition, and it is associated with poor health outcomes.

A multitude of environmental, metabolic, and endocrine effects can cause IUGR and SGA. Current research to establish better ways to determine which infants are pathologically small might help us to prevent perinatal morbidity and mortality [22]. Recently, genes in SGA infants, which are related to B cell development, metabolism, and obesity, were found to be hypomethylated compared to those in non-SGA infants [23]. Thus, DNA methylation profiles may prove to be a useful diagnostic tool if epigenetic changes are indeed the key mechanism underlying DoHAD and the pathogenesis behind SGA. 

In addition, maternal nutrition and nutrient transport across the placenta directly influence fetal development [24]. Factors that influence the transfer of substances between maternal and fetal circulations are numerous, including utero-placental and umbilical blood flow, concentration gradients of nutrients, thickness of the exchange area, and metabolism of the placenta. However, what are the mechanisms by which the environment can influence early developmental programming and resultant adult disease? The key lies in epigenetic DNA and histone modifications. 

## 4. Epigenetic Mechanisms and Fetal Development

Every cell in the human body and in the developing embryo has a unique epigenome, which determines which genes are expressed and by how much. Epigenetic modifications encompassing the epigenome can be relatively stable while transmitting from one cell generation to the next. DNA methylation, histone modifications, genomic imprinting, chromatin remodeling, and non-coding RNA are all mechanisms for epigenetic modifications. DNA methylation of cytosine bases and histone modifications cause specific changes in chromatin structure and therefore regulate gene expression patterns. DNA methylation is catalyzed by DNA methyltransferase (DNMT). Methylation to nucleotide bases at cis-acting elements, such as promoters or enhancers, cause DNA conformational changes that prevent the binding of trans-acting factors which initiate gene expression. Histone modifications can alter the charge differential between the histone tail and DNA double helix and, thus, change in chromatin compaction. Examples of histone modifications include methylation, acetylation, phosphorylation, and others. Different types of enzymes catalyze each type of histone modification. For example, histone acetyl transferases (HATS) histone methyltransferases catalyze acetylation modifications to histones and methylation, respectively, while other enzymes remove these marks (HDACS). Genomic imprinting, a parent-of-origin-specific silencing of certain gene alleles established in the germline (sperm or egg cells), is maintained throughout the lifetimes of cells. Short RNAs such as miRNA and siRNA and recently discovered tRNA fragments are all involved in epigenetic processes [25].

The pre- and peri-implantation period of embryonic development is a sensitive window when cells are most vulnerable to changes in epigenetic programming. Historically, it was believed that global demethylation occurred in two distinct phases during embryo development. The first phase involved a global, active round of demethylation of the paternally inherited genome, occurring within the first cell cycle after fertilization. In contrast, the maternally inherited genome was believed to lose methylation progressively by a failure to replicate the methylated state following multiple cell divisions. By the blastocyst stage, demethylation was established, followed by a marked round of methylation after implantation. More recently, the loss of methylation from the paternally derived genome was shown to be incomplete, such that demethylation occurs, but more to a level similar to that observed in the hypomethylated maternally inherited genome [25]. Furthermore, newer studies detected no evidence for a global failure of maintenance methylation in the maternal genome [25]. Rather, following compaction, there is significant loss of methylation from the cells that go on to form the ICM (inner cell mass). Then, following implantation, methylation patterns increase on a global genome-wide scale [26]. 

The methylation state of only 6.8% of CpGs in the genome remains stable from that in sperm to cells in the ICM of blastocysts on Day 7.5 of development [27]. The most stable CpGs are largely unmethylated in sperm and oocytes, and a smaller number of stably methylated CpG are mainly in nucleotide repeats or introns. For most CpGs, the average methylation levels are higher in sperm than oocytes. Additionally, enhancer elements are demethylated in ICM cells [27,28]. Knowing that the epigenome becomes increasingly methylated throughout development, what is driving these epigenetic changes? 

The following sections describe some ways epigenetic reprograming can be altered in early development, including changes in amino acid transport, dietary protein, and extracellular vesicles. 

## 5. Possible Roles of Amino Acid Transport and Metabolism in Epigenetic Changes

Amino acid transporters influence pre- and peri-implantation embryo development in numerous ways. Since these effects occur early in development, when embryos undergo quantitatively large amounts of epigenetic reprograming [29], they likely involve some pivotal qualitative DNA and histone modifications that may be transgenerational. First, proline (Pro) uptake promotes early embryo development both after the two-cell stage and the during fertilization of mouse oocytes in vitro [30,31]. While the presence of Pro during fertilization fosters subsequent blastocyst formation, there are not as many trophoblast cells in the blastocysts as compared to blastocysts formed in vivo. The latter cell number should be a goal to produce the healthiest embryos in vitro and offspring after embryo transfer to the reproductive tract. 

Similarly, in vitro culture with Pro during cleavage promotes the formation of blastocysts in a growth factor-like manner, both when embryos are cultured at high and low density and even in hyperosmotic oviductal fluid-like medium [30]. Pro may act on embryos via Pro transporter(s) as transceptor(s) and the signaling of this nutrient inside cells. mTOR1, Akt, and ERK are all activated by Pro transport into these cells [30]. By studying the mechanisms of this signaling further, we might develop better culture media and, thus, reduce adverse pregnancy outcomes and health risks to future generations. 

Gln transport into early embryos also promotes their development into blastocysts in isosmotic and especially in hyperosmotic media at both high- and low-density embryo concentrations [30]. As for Pro, the production of the healthiest conceptuses in vitro likely requires Gln in culture media. Such measures should then promote the development of healthy adults following embryo transfer to the reproductive tract. 

The transport of glycine (Gly) into cleavage stage embryos protects them from otherwise the detrimental effects of the hyperosmotic medium designed to resemble oviductal fluid [32,33]. Gly serves as an intracellular osmolyte owing to its Na^+^-dependent transport against total chemical potential and concentration gradients [32,33]. Thus, physiologically normal hyperosmotic oviductal secretions likely interact with their normal concentration of about one mM of glycine [34] to promote cleavage-stage embryo development. We have yet to learn whether embryos and resultant offspring become healthier the more we mimic, for assisted reproduction in vitro, the physiological conditions of early embryo development in vivo. 

System B^0,+^ is novel in its transport of both zwitterionic and cationic amino acids into blastocysts, although it especially prefers branched chain and benzenoid amino acids, such as leucine (Leu) and tryptophan (Trp) [35]. In species where blastocysts invade the uterine epithelium, B^0,+^ promotes implantation and further development owing to Leu-stimulated mTOR1 signaling [35,36,37,38,39]. Since a maternal LPD during preimplantation development results in a Leu concentration decrease of about 25% in uterine secretions [40], Flemming and associates proposed that mTOR1 signaling would also be decreased [41,42]. In this model, increased trophoblast pinocytosis and lysosome biogenesis compensate to provide blastocysts and later stages greater histotrophic nutrition. The resultant offspring grow faster, have more adipose tissue, and may eventually have bone alterations, neural dysfunction, hypertension, and cardiovascular abnormalities [41,42], likely owing to disturbances in epigenetic histone and DNA modifications.

The Na^+^-independent system B^0,+^ in mouse blastocysts also transports both cationic and zwitterionic amino acids [43]. However, arginine (Arg) is the strongly preferred amino acid by this transporter [39,44,45]. Studying system B^0,+^ in blastocysts helped to reveal multiple ways to foster trophoblast motility [35,36,37]. In addition to Leu, Arg by itself activates mTOR1 and, thus, fosters blastocyst development and trophoblast motility [36]. Moreover, polyamines and nitric oxide are both products of Arg metabolism, and they too promote cell motility [35]. Owing to this redundancy, the knockout of either system B^0,+^ or system B^0,+^ does not noticeably affect early embryo development or the fertility of mouse offspring [38,46]. Nevertheless, prenatal dietary supplementation with Arg promotes fetal growth in complicated pregnancies [47]. 

For the blastocyst inner cell mass (ICM), embryonic stem (ES) cells are used as models. According to studies with mouse ES (mES) cells, ICM cells take up threonine (Thr) via at least three obligate exchange transporters–two Na^+^-dependent ASC transporters and one Na^+^-independent system L [48]. While Thr uptake was first found to be important for its metabolism (see below) [49], its transporter(s) may also be transceptor(s) [50] that help maintain proliferation and pluripotency. In support of their function as transceptors, we predicted correctly that the threonine analog, 3-hydroxynorvaline (3-HNV), would block human ES (hES) cell proliferation even though the cells do not metabolize Thr in the way mES cells do [51]. Altered Thr signaling in ICM cells owing to, say, a maternal LPD and the resultant lower competition for uptake by asparagine [40,48], might influence the subsequent development of tissues and organs by ICM cells.

Except for humans, mammalian ES cells likely need Thr metabolism to remain pluripotent and proliferate [49,52]. In mES cells, threonine dehydrogenase (TDH) catabolizes Thr to Gly and acetyl CoA. The Gly generated by TDH is used specifically to produce one carbon (1C) methyl groups needed for the trimethylation of Lys residue 4 in histone 3 (H3) and maintenance of mES cell pluripotency [53,54]. However, humans produce an inactive TDH protein and likely obtain the Gly needed for H3K4me3 formation in hES and ICM cells by upregulating serine (Ser) synthesis from glycolytic intermediates [54]. Serine hydroxymethyltransferase then converts this Ser to Gly. 

Finally, removal of Lys from the culture medium nearly blocks hES and likely ICM cell proliferation [55]. Deprived of Lys uptake, hES cells appear to be unable to produce a specialized pool of glutamate (Glu) needed to bind to Glu receptors and maintain pluripotency [56]. While mES cells are less reliant on Lys in the medium for Glu production [49], they still need Glu signaling via their metabotropic Glu receptors to continue to proliferate and remain pluripotent [57,58]. Moreover, maternal LPD consumption causes a decrease in the Lys concentration in mouse blastocysts [40] where exogenous Glu for the ICM may be in shorter supply than in mES cell culture medium. Hence, decreased Glu production from Lys in ICM cells may be one mechanism by which LPDs alter early embryo development and produce unwanted health consequences in adults [38,39,41,42,59]. 

## 6. Nutrient Deficiencies Alter Early Development

The maternal diet during pregnancy can alter one-carbon metabolism and cause FGR. Amino acids, such as methionine, serine, and glycine, are needed not only as substrates for protein synthesis during pregnancy but also for the one-carbon metabolism affecting stem cell proliferation, differentiation, and, subsequently, the health of fetal tissues and organs. Methionine and folate (Vitamin B9) are key constituents of one-carbon metabolism, which provides one-carbon units for methyl-transferase reactions. These molecules provide substrates for DNA and histone methylation, so changes in nutrient availability, such as dietary protein or folate deficiencies, could have significant impacts on the epigenetic programing of the developing embryo. (See also the section on amino acid transport above).

For example, a maternal LPD in rats decreases methylation of the PPARα gene and glucocorticoid receptor genes in their offspring. This increases the expression of the PPARα gene and glucocorticoid receptor genes and can be prevented by folic acid supplementation [60]. Increased expression of these genes is associated with metabolic and cardiovascular (CV) disturbances. Additionally, LPDs cause the upregulation of genes implicated in adipocyte differentiation and metabolism [61]. Maternal malnutrition also results in epigenetic alterations of several genes related to hypertension [62], regulation of food intake, and glucose homeostasis [63].

A low maternal folate status during pregnancy is associated with increased risks of pregnancy complications, intrauterine growth restriction, and developmental disorders including learning disabilities and emotional problems. In addition, polymorphisms in the MTHFR gene (coding for methylene tetrahydrofolate reductase, the rate limiting enzyme in methyl group production) likely contribute to the risk of developing psychiatric disorders, including depression, schizophrenia, and bipolar disorder [64].

Hence, alterations in folate availability during pregnancy lead to variations in DNA methylation patterns in the offspring, which sometimes change gene expression and the outcomes of disease [65]. For example, folate deficiency in early pregnancy results in dysregulated 1-C metabolism, as revealed by an overall lower DNA methylation level. The maternal CpG sites, whose methylation levels change as the pregnancy proceeds, are most likely influenced by alterations in maternal folate and one-carbon metabolite concentrations [66].

Most studies focus on transgenerational characteristics passed to offspring from mothers, but recent studies suggest that the father plays a role as well. For example, altered sperm miRNA and tsRNA have been observed following paternal exposure to diet change or stress [67]. Moreover, the injection of sperm RNA from males exposed to an unhealthy diet or stress into early embryos results in transgenerational inheritance in mammals [68]. Hence, sperm RNAs are likely active epigenetic modulators of offspring phenotypes, and small RNAs in sperm are altered following changes in male nutrition. Grandjean et al. showed that the microinjection of either testis or sperm RNA from male mice fed a Western-like diet into one-cell embryos results in offspring with a metabolic phenotype associated with a Western-like diet. In contrast, RNAs from male mice fed a healthy diet did not produce offspring with an undesirable metabolic phenotype. More specifically, they found that several microRNAs had increased expression in healthy versus sick controls, and when they microinjected the microRNA miRNA19b into one-cell embryos, this induced metabolic alterations that are similar to the diet-induced phenotype [69]. 

Follow-up studies conducted by Sharma et al. used an LPD-based mouse model and observed a metabolic phenotype, a decreased cholesterol ester level, in the offspring of males that consumed an LPD [70]. Consistent with the previous study, they report altered miRNA levels in sperm of these males. Subsequently, however, they focused on tRNA fragments, which are the most abundant class of RNA in mature sperm and were postulated to be most functionally relevant. To investigate this possibility, they used antisense oligonucleotides against the specific tRNA fragment of interest. In association with tRNA fragments from mice consuming an LPD, they saw the upregulation of several genes in offspring that are targets of the endogenous retroelement MERVL [70]—an incredibly interesting finding since endogenous retroelements have been linked to multiple disease states in humans, including metabolic syndrome, autoimmunity, and cancers [71]. 

## 7. Role of Extracellular Vesicles in Epigenetic Changes during Early Development

Extracellular vesicles (EVs) can be an additional source of protein and other biomolecules that have the potential for influencing cell-to-cell communication throughout development in utero. Membrane-bound extracellular secretory vesicles convey information between cells through the transfer of functional protein and genetic information to influence the function and phenotype of recipient cells. EVs influence oogenesis, oocyte maturation, fertilization, and crosstalk between mother and embryo/fetus. In addition, EV-mediated communication in the female reproductive tract occurs between seminal plasma EVs and the endometrium, oviductal tract EVs and both sperm and embryo, follicular fluid EVs and embryo, embryo EVs and the endometrium, placenta EVs and extravillous trophoblasts, and endometrium EVs and the embryo [72]. Approximately 3500 different proteins have been isolated from EVs based on the cell type [73]. The functions of EV cargo range from the modulation of sperm capacitation to the regulation of embryo development and implantation [73]. Moreover, the placenta has been shown to secrete EVs carrying proteins that are involved in immune modulation and critical for a successful pregnancy [74]. In addition to proteins, EV microRNAs function in intercellular communication during pregnancy through the modulation of gene and protein expression. Studies have shown that both the mother and baby produce EVs that rely on one another for successful development. Extracellular vesicles originating from the embryo are taken up by human primary endometrial epithelial and stromal calls [75] and endometrial EVs, and their miRNA cargo are internalized by embryos [76]. The RNAs present in these extracellular vesicles may provide necessary instructions for epigenetic changes and allow room for even more genetic diversity in the offspring, based on the early environment conditions on both the paternal and maternal side. 

## 8. Implications of Assisted Reproductive Technology (ART) to the Barker Hypothesis

As discussed above, EVs play an important role in embryo implantation, fetal development, and even the transgenerational transmission of pathology. Important considerations should include any intervention that may induce stress from the peri-implantation period through delivery. Sources of physiologic fetal stress, including malnutrition or overnutrition, have been linked with poor adult outcomes, first according to the Barker hypothesis and then through multiple studies, many of which were just discussed. 

While ART has helped many infertile couples conceive, the procedure and culture medium are also a source of stress on the embryo. In an advanced age mouse model, the supplementation of the embryo culture medium with EVs derived from endometrial mesenchymal stem cells improved the embryo’s developmental competence and the total blastomere count [77]. Recently, Asaadi et al. demonstrated that supplementing in vitro maturation media with EVs from ampullary oviduct fluid was beneficial for bovine embryo development and quality [78]. Conversely, EV cargo such as miR-661 has been found in higher quantities in the culture media of IVF embryos that did not implant, suggesting some may have anti-implantation properties. As expected, miR-661 downregulates genes involved in adhesion and therefore likely inhibits implantation [79]. Apart from affecting implantation, ART is also associated with an increased risk of poor outcomes including birth defects [80] and imprinting disorders [81].

Several studies have demonstrated that independent of gestational age and ART method, ART pregnancies result in a significantly lower birth weight, and a higher placental weight [82]. In 2015, Chen et al. confirmed these findings using a mouse model. Additionally, researchers found that ART manipulation can cause defects in placental layer segregation, glycogen cells migration, and the expression of placental nutrient transporters including amino acids and glucose. They discovered several significantly downregulated imprinted genes including IGF2, which has been shown to play a vital role in regulating glycogen cell development and placental nutrient transporter expression [83]. Katari et al. (2009) were first to report a cumulative analysis of CpG methylation differences between children conceived via ART and children born via natural conception [84]. In vitro conception was found to be associated with lower mean methylation at CpG sites in the placenta and higher mean methylation at CpG sites in cord blood. Interestingly, they also found that in vitro conception-associated DNA methylation differences are associated with gene expression differences at both imprinted and non-imprinted genes. Several genes associated with adipocyte development, insulin signaling, and obesity (CEBPA, MEST, NNAT, and SERPINF1) have significantly lower mean transcript levels in children conceived in vitro. Similarly, compared to placentas formed after natural conception, IVF/ICSI placentas exhibit increased H19/IGF2 and SNRPN gene expression, owing to their decreased methylation [85,86,87]. 

These DNA methylation defects shown in ART pregnancies are largely dependent on the availability of methyl groups, which is diminished in one-carbon metabolism deficiency. Using a mouse model, Rahimi et al. 2019 demonstrated that moderate folic acid supplementation in ART resulted in beneficial effects when compared to a control diet [88]. These effects included decreased embryonic developmental delay, reduced DNA methylation at certain ICRs, and increased global DNA methylation in embryonic and placental tissues. Importantly, high-dose folic acid supplementation had detrimental effects, suggesting a dose dependency, which has been observed previously in mice [89,90]. High folic acid supplementation leads to elevated levels of unmetabolized folic acid in the circulation, which may contribute to the observed deleterious effects by downregulating critical enzymes in one-carbon metabolism [91].

## 9. Conclusions

Determining the existence of transgenerational epigenetic effects can be challenging. Not only do humans have a long gestational period, but the human lifespan makes it difficult to assess transgenerational effects. It is also difficult to determine if the phenotype is caused from transgenerational inheritance or was acquired post conception. It is difficult to separate the epigenetic effects of trauma on offspring who lived through the trauma from the effect of growing up with a parent who lived through severe trauma. Working with animal models in the lab environment allows for controlled studies to isolate the exposure, whereas these types of studies with humans are very difficult to control. 

A possible innovative way to study transgenerational epigenetic marks could be through building on recent advancements in stem cell technologies. Researchers are currently working on ambitious efforts to convert adult stem cells into gametes [92]. Success has been achieved in mice but not yet in humans [92]. Since we are already having success with animal models, it is a matter of time before we are able to produce gamete cells from stem cells. Jamie Metzel, in the book *Hacking Darwin*, presents a possible future way to study transgenerational effects by producing multiple generations of embryos in a short period of time. In vitro fertilization can then be performed, and a particular embryo is selected, but rather than implanting that early stage embryo in the mother, the cells can be extracted to make new germ cells in the form of eggs [93]. One could imagine changing the environmental conditions that the initial germ cells or embryo is exposed to, such as low protein or high protein in the media, and then testing the transgenerational effect of those contexts. 

The Barker hypothesis has been supported by a multitude of epidemiological and retrospective studies that show changes in early life can predispose an individual to adult-onset diseases such as metabolic syndromes, obesity, cardiovascular diseases, and even cancer. Low birth weight has been correlated to an increased susceptibility to the development of some of these disorders. The mechanism behind these changes is thought to be at least in part due to changes in the epigenome following these variable environmental conditions that persist into adulthood. Fluctuations in one-carbon metabolism, amino acid transporter activity, dietary changes, and the formation of extracellular vesicles or exosomes have all been shown to affect epigenetic marks that translate into human disease phenotypes. Further research is needed to elucidate the full scope of early environmental challenges and their associated transgenerational epigenetic impact. 

## Data Availability

No new data are reported in this review.
